# Using chills-inducing music to augment self-transcendence, emotional breakthrough, and psychological insight during mindfulness and loving kindness meditation

**DOI:** 10.3389/fpsyg.2026.1589132

**Published:** 2026-02-03

**Authors:** Leonardo Christov-Moore, Felix Schoeller, Mathilda Von Guttenberg, Tiffany Durinski, Mordechai Walder, Felipe A. Jain, Marco Iacoboni, Nicco Reggente

**Affiliations:** 1 Institute for Advanced Consciousness Studies, Santa Monica, CA, United States; 2 Department of Psychiatry, Massachusetts General Hospital, Harvard Medical School, Boston, MA, United States; 3 Harvard-MIT Division of Health Sciences and Technology, Boston, MA, United States; 4 Ahmanson-Lovelace Brain Mapping Center, UCLA, Los Angeles, CA, United States

**Keywords:** technodelics, non-ordinary states, meditation, loving kindness, chills

## Abstract

**Introduction:**

Non-pharmacologically induced altered states of consciousness that promote mental health and wellbeing are a growing focus of clinical and basic research. Previous work has revealed the mood-augmenting, belief-altering, and self-transcendent effects of aesthetic-chills-inducing audiovisual stimulation. The current study investigated how a guided loving kindness meditation (LKM) combined with uplifting, chills-inducing music (henceforth: chills-augmented) affected participants’ mood, self-transcendence (ST), psychological insight, and emotional breakthrough.

**Methods:**

We conducted a randomized, controlled online study (*n* = 398) using a 2 × 2 design comparing a validated loving kindness meditation (LKM) to mindfulness-based control (MC), each with chills augmentation (+) and without (−).

**Results:**

As hypothesized, LKM, compared to MC, increased connectedness to others, while chills augmentation to either stimulus (LKM+/MC+) enhanced ST, mood, emotional breakthrough, and psychological insight. Mediation analyses confirmed that the occurrence of aesthetic chills during meditation predicted these downstream effects. They also found trait measures that independently (of main effects) contributed to distinct outcomes: absorption predicted feelings of ego-dissolution, connectedness to the world and self, and moral elevation; interoceptive awareness predicted ego-dissolution and connectedness to self; and vividness of internal imagery predicted connectedness to the world and others.

**Discussion:**

Chills augmentation appears a viable method for enhancing the immersiveness, salience, and downstream positive impact of guided contemplative interventions, without interfering with the intended outcome. This work can further our understanding of and access to non-ordinary experiences that beget salutogenic, prosocial outcomes.

## Introduction

Non-ordinary or altered states have experienced a revived interest as a promoter of individual well being, meaning-making and social cohesion (Franco Corso et al., 2023). Self-transcendence (ST) —characterized by elevated mood, ego-dissolution, interconnectedness, and moral elevation— is one such state with significant clinical potential (Ko et al., 2022). Research consistently links higher propensity for ST to improved mental health outcomes, including reduced depression (Haugan and Innstrand, 2012), enhanced self-esteem, and stronger internal locus of control (Ellermann and Reed, 2001; Er and Buzlu, 2022). This relationship has been documented across numerous studies (Abu Khait et al., 2020; Coward, 1996; Reed and Haugan, 2021; Bovero et al., 2023; Hwang et al., 2019; Liu et al., 2021; Pizarro et al., 2021; Runquist and Reed, 2007; Thomas et al., 2010). ST has proven valuable during challenging life events, helping people find coherence, purpose, and resilience (Nygren et al., 2005), and has shown promise in helping people cope with uncertainty, such as during the COVID-19 pandemic (Worth and Smith, 2021; Wong et al., 2021). The occurrence of ST also mediates positive attitudes toward others, following experiences like awe (Jiang and Sedikides, 2022; Li et al., 2019), nature immersion (Castelo et al., 2021; Neill et al., 2019), meditation (Miller and Verhaeghen, 2022), and social activism (Barton and Hart, 2023).

While ST has empirically measurable features, it is rooted in Buddhist teachings on *anattā* (non-self) and *pratītyasamutpāda* (dependent origination), which describe the dissolution of rigid self-boundaries and recognition of fundamental interconnectedness as pathways to spontaneous compassion (Bodhi, 2000). For over two millennia, Buddhist meditation practices have served as systematic methods for experientially realizing these theoretical insights, with contemplative practitioners using techniques like mindfulness, concentration, and loving-kindness cultivation to induce the states of ego-dissolution, interconnectedness, and moral elevation now measured in contemporary ST research (Perry et al., 2025). Loving kindness meditation (LKM) is one such traditional Buddhist approach, designed specifically to cultivate compassion through the progressive expansion of loving awareness from self to all beings, thereby operationalizing the theoretical principle that wisdom naturally gives rise to universal care when the boundaries of separate selfhood are transcended (Salzberg, 2019). LKM presents a tractable, validated path to cultivate ST toward mental health and wellbeing (Hofmann et al., 2011), with established neural mediators (Mascaro et al., 2015). Loving kindness meditations include: Metta, a guided imagery practice focused on expanding the circle of self (Frick et al., 2020); Tonglen, in which the breath is used to cultivate compassion and transmit kindness to others (Chödrön, 2001); among others. LKM has been shown to enhance markers of ST, empathic concern, and prosociality (Bankard, 2015; Luberto et al., 2018; Sacchet et al., 2024). Although loving kindness meditation is ostensibly uncomplicated and requires no special equipment beyond dedicated adherence to a contemplative style, cultivating these states often demands sustained attention, mental discipline, and consistent practice—challenges that can be particularly pronounced without the guidance of a teacher (Brandmeyer et al., 2023; Lumma et al., 2015; Kropp and Sedlmeier, 2019). This introduces an additional barrier in efforts to democratize these experiences. While meditation apps and online videos have helped increase the accessibility of loving kindness practices (Jiwani et al., 2023), additional forms of augmentation may both aid adoption by novices and enhance these meditations’ downstream effects.

Recent research points to aesthetic chills (also known as “moving chills”; Bannister, 2019; hereafter “chills”) as a promising method to increase ST (Christov-Moore et al., 2024). These pleasurable psychophysiological responses—marked by cold sensations, shivering, and goosebumps (Benedek and Kaernbach, 2011)—are universally recognized, replicable, and achievable within brief time windows. Chills can occur in response to various stimulus types including art, music, scientific lectures, and religious content (Schoeller, 2015). Individual susceptibility to these experiences varies, correlating with personality traits like openness and absorption (McCrae, 2007; Schoeller et al., 2023a, 2023b; Silvia and Nusbaum, 2011). Importantly, these aesthetic experiences appear to enhance mood, awe, and prosociality (Fukui and Toyoshima, 2014; Jain et al., 2023; Haar et al., 2020), mirroring outcomes associated with traditional ST experiences (Christov-Moore et al., 2024; Schoeller et al., 2023a, 2023b). Chills represent a promising, replicable marker of aesthetically-evoked ST, complete with identifiable physical and neurophysiological markers (Benedek and Kaernbach, 2011; Sumpf et al., 2015). As peak experiences, they follow a characteristic temporal sequence: from initial anticipation (“wanting”), through the consummatory pleasure of the chill itself (“liking”), to a subsequent learning phase where meaning is consolidated (Schoeller et al., 2024a). Although chills are associated anecdotally with spiritual insight and practice (“spiritual chills”)—perhaps due to this meaning-making characteristic—there is scant evidence for chills as a component of contemplative practice, with the notable exception of Kundalini practitioners (Maxwell and Katyal, 2022).

These lines of evidence—alongside theoretical advances suggesting that both meditation and peak experiences may enhance psychological “empowerment” and flexibility by operating at critical points between rigidity and chaos (Atasoy et al., 2019)—raise an intriguing possibility for enhancing contemplative practice: if both meditation and chills can independently induce ST through complementary mechanisms, could their combination create synergistic effects? This cybernetic perspective suggests meditation could provide stable regulatory foundations while chills create opportunities for rapid state-shifts and meaning making, potentially offering a powerful pathway to psychological wellbeing. We investigated this potential synergy by examining whether loving kindness meditation naturally evokes chills, and whether deliberately augmenting meditation with chills-inducing stimuli could amplify ST experiences and their beneficial outcomes.

Leveraging both an LKM and aesthetic-chills-inducing musical soundtrack, we sought via their combination to create a reliable, non-pharmacological experience to induce ST, with an emphasis on connectedness to others. Furthermore, given the meditation’s incorporation of body-scanning practices, narrative elements, and visual imagery we sought to elucidate the roles of trait interoceptive awareness, absorption, and internal imagery in facilitating intervention outcomes, and investigate the mediating role of aesthetic chills in increasing the self-transcendent, meaning-making aspects of guided meditations.

Our hypotheses were as follows:

*H1:* LKM meditation causes increased connectedness to others relative to a mindfulness-based control.

*H2:* Chills augmentation increases likelihood and intensity of self-reported chills.

*H3:* Chills occurrence mediates ST outcomes resulting from Chills augmentation.

*H4:* Vividness of internal imagery (VVIQ) moderates LKM effects on connectedness to others.

*H5:* Interoceptive awareness (MAIA) moderates LKM effects on chills occurrence.

## Method

We report how we determined our sample size, all data exclusions (if any), all manipulations, and all measures in the study.

### Participants

Participants (*N* = 416) were recruited through Prolific, an online platform with comprehensive pre-screening features commonly used to recruit participants (Palan and Schitter, 2018). Prolific is a specialized online platform designed to connect researchers with a global pool of participants for research studies, offering tailored participant recruitment through a range of pre-screening tools including mental health diagnoses, medication, age, gender identity, nationality, and employment status. Participants were English-speaking U.S. residents with no hearing difficulties or history of neurologic disorder. The platform was instructed by the research team to ensure an approximately equal proportion of male and female participants. After data quality control (see below), 18 participants were removed and *N* = 398 participants remained in our total sample for analysis.

### Sampling

Each of the four conditions was prepared as a separate, otherwise identical survey, titled “Meditation Study” 1 through 4. Online participants then chose to conduct one or another iteration of the survey (the only difference being the stimulus) but were excluded from participating in further surveys within the set once one had been completed. Since no information on the conditions was available from the title, this accomplished semi-random sampling within the confines of Prolific’s overall study ecosystem.

Prolific’s general study sampling approach offers some notable advantages, primarily being more demographically diverse than typical laboratory samples. While university research often relies on undergraduate students aged 18–23, Prolific’s participant pool encompasses a broader age range and varied education and employment backgrounds.

### Design

We employed a 2 × 2 design comparing LKM to a mindfulness-based control meditation (MC), with and without musical augmentation designed to elicit chills.

## Materials

### Meditation stimuli

LKM: The study’s first and senior authors developed a custom guided meditation, synthesizing Metta, Tonglen, and body scanning practices, drawing inspiration from Salzberg (2019), Chödrön (2001), Goenka (2019), and mentalizing imagery therapy (Jain and Fonagy, 2020). The meditation establishes the cycles of inhalation and exhalation as attentional anchors, using these natural rhythms to frame specific contemplative instructions. Participants are guided to use each breath cycle as a framework for cultivating compassion—inhaling acknowledgement and empathy then exhaling kindness—while progressively expanding their circle of attention from all aspects of the self, through loved ones, and ultimately to all living beings, connected through their shared experience of (c.f., umwelt), or encounter with, suffering and impermanence. The total duration of the meditation was 28 min. It was accessed via an embedded link within a devoted page of the survey, which participants were required to complete before proceeding to the next page. The script and audio recording were iteratively revised several times prior to the study based on feedback from leaders in meditation research and several therapists (see acknowledgments).

MC: As a putative mindfulness meditation control, we used an existing mindfulness-based meditation, “Waking Up with Sam Harris” (Harris, 2023). In it the participant is invited to focus on the breath and on the objects of experience, and reflect on how when the self is sought within conscious experience, it becomes apparent that what we identify as the “self” is experience itself. The meditation encourages non-judgmental awareness of non-duality, without any explicit invitations to cultivate empathy or kindness. The total duration of the meditation was 26 min. It was accessed via an embedded link within a devoted page of the survey, which participants were required to complete before proceeding to the next page.

### Musical augmentation for chills induction

Drawing from empirically-validated musical stimuli known to reliably induce aesthetic chills in a large percentage of participants (*Chills DB 2.0*; Schoeller et al., 2023a, 2023b), we selected five of the most potent chills-inducing musical pieces to augment both meditation conditions. These validated stimuli were systematically integrated into the LKM and MC narratives, in the form of a soundtrack, which frequently lay under the spoken narrative. Segments were positioned to align with narrative peaks while maintaining equivalent temporal placement across conditions to ensure controlled comparison of augmentation effects. These manipulations yielded four experimental conditions in a 2 × 2 factorial design: meditation type (MC/LKM) × chills augmentation (+/−). Professional audio engineering services for post-production and mastering were provided by Epitone Studios (Las Vegas, NV).

### Combined audio files

The full stimuli can be experienced by the reader at the following url’s:

LKM(+): https://www.youtube.com/watch?v=Id-zXX7ac-o
LKM(−): https://www.youtube.com/watch?v=R3i3sUywdTo
MC(+): https://www.youtube.com/watch?v=Nxjx6_zYX-4
MC(−): https://www.youtube.com/watch?v=CN-_zzHpcdM

### Pre-intervention measures

#### Demographic information

Participants provided their age, gender identification, political orientation (1–7, very liberal to very conservative), meditation experience (1–10 no experience to highly experienced), and religiosity (7-item centrality of religion and spirituality index [CRSi-7]; Huber and Huber, 2012).

#### Dispositional positive emotion scale (DPES)

The *DPES* (Shiota et al., 2006) measures one’s dispositional tendencies to feel positive emotions towards others in their daily lives.

#### NEO five-factor inventory (NEO-FFI-3)

The *NEO-FFI-3* (Costa and McCrae, 1992) is a widely used personality assessment tool measuring five broad dimensions: *neuroticism*, *extraversion*, *openness to experience*, *agreeableness*, and *conscientiousness*.

#### Modified Tellegen absorption scale (MODTAS)

We employed a modified version of the *Tellegen Absorption Scale*, which has a Likert-scaled response format and a clearer covariance structure than the original *TAS* (Jamieson, 2005).

#### Kama muta frequency scale (KAMF)

This 7-item scale (Zickfeld et al., 2019) measures predisposition for *Kama muta*, (काममूत in Sanskrit, meaning: “moved by love”) an affective state described as “being moved”, “heart-warming”, “stirring”, or “being emotionally touched”.

#### Multidimensional assessment of interoceptive awareness (MAIA)

The *MAIA* is a comprehensive self-report instrument designed to measure interoceptive body awareness (Mehling et al., 2018).

#### Vividness of visual imagery questionnaire (VVIQ)

The *VVIQ* consists of 16 items in four groups of 4 items in which the participant is invited to consider the mental image formed when thinking about specific described scenes and situations. The vividness of the image is rated along a 5-point scale (Marks, 1973).

### Pre/post-stimulus affective state assays

We administered a brief, 10-item set of measures to assess emotional valence and arousal. Valence and arousal were each measured using single-item ratings, a common practice supported by well-established frameworks like the *Self-Assessment Manikin* (Bradley and Lang, 1994) and the circumplex model of affect (Russell, 1980). Although single-item measures do not yield traditional reliability indices, they are frequently employed in experimental settings due to their strong face validity and low participant burden.

### Post-intervention measures

#### Chills self-report

Following standard procedure in the literature, chills were self-reported by the participants through a series of questions regarding their emotional and physiological responses to the stimulus. They responded to binary (Yes/No) questions such as “Did you experience chills?” and “Did you experience goosebumps?”, as well as questions about the frequency and intensity of chills rated on a 0–10 Likert scale.

#### Ego-dissolution inventory

The *EDI* (Nour et al., 2016) consists of sixteen items relating to altered ego-consciousness, eight relating to the experience of ego-dissolution (comprising the EDI), and eight relating to the antithetical experience of increased self-assuredness, termed ego-inflation, rated using a visual analog scale ranging from 0 to 100%.

#### Watts connectedness scale

The *WCS* (Watts et al., 2022) measures connectedness to self (e.g., “My mind felt connected to my heart/emotion.”), connectedness to others (e.g., “I felt connected to friends and/or family.”), and connectedness to the wider world and spirituality (e.g., “I felt that everything is interconnected.”), rated using a 1–5 Likert scale.

#### State moral elevation scale

The *SMES* (McGuire et al., 2022) assays Emotional Reaction (“in touch with the better parts of myself”), Physical Reaction (“a warm or glowing feeling in my chest”), and Motivation (“motivated to live in a nobler or virtuous way”) rated using a 1–5 Likert scale.

### Procedure

Participants in the survey were first instructed to respond as to whether they would conduct the questionnaire in good faith, a measure recommended by the Prolific platform to increase completion and produce more reliable responses. Participants then reported demographic information and completed the pre-questionnaires. Participants were instructed to report their affective state prior to receiving methodological guidance: identifying a conducive, undisturbed environment, closing their eyes, and preparing to engage with the experimental stimulus.

After listening to the meditation, participants reported their affective state again and were asked about their degree of immersion (i.e., extent of engagement throughout the meditation), whether they experienced chills or tearfulness, and the intensity of these experiences. Three measures of self-transcendence were collected: ego-dissolution, connectedness, and moral elevation. They additionally reported on psychological insight and emotional breakthrough. Open text fields allowed participants to report what aspects of the meditation gave them chills or caused tearfulness, as well as any general comments about the experience.

### Ethics statement

Using the Department of Health and Human Services regulations found at 45 CFR 46.104(d)(3), the Advarra IRB (Columbia, MD) determined that this research project (Pro00079627) is exempt from IRB oversight after reviewing the project’s protocol, recruitment materials, and related questionnaires dated May 14, 2024.

### Data quality control

Based on in-house piloting, which determined that comprehensive question response requires 5–7 min while maintaining comprehension, and given the 26–29 min meditation duration, we established a minimum survey completion threshold of 33 min. Participant responses falling below this temporal benchmark were deemed unreliable, systematically excluded, and participants were requested to withdraw their response (as rejected responses can affect participants’ user ratings). These responses are not considered within the total *N*, with the observed average survey completion time being 45 min.

Using a procedure from prior studies, we removed participants who reported experiencing chills yet indicated zero chills intensity, or who reported no chills but indicated chills intensity over three, as these indicated failure to understand instructions. A total of *N* = 398 participants remained: 99 in the MC(−) group, 91 in the MC(+) group, 110 in the LKM(+) group, and 98 in the LKM(−) group. A power analysis determined that for the principal analyses, our total sample size of 91 minimum participants per group, even following exclusions of 18 people altogether, was sufficiently powered to detect even a relatively small main effect of meditation type, chills augmentation, or interaction effect (~0.19) on chills intensity in the context of an ANCOVA.

### Analysis

We first conducted descriptive statistical tests on all analyzed measures, finding approximately normally distributed responses via observation of Q-Q plots. Given our large sample size (*n* > 30 per group), we felt further justified in using parametric analyses via the central limit theorem. In order to predict chills occurrence (which can only take on two values, 0 = no, or 1 = yes), we conducted a binomial logistic regression. To examine main effects and interaction effects driving chills and self-transcendent outcomes, as well as emotional breakthrough and psychological insight, we conducted a series of analyses of covariance (ANCOVA) using chills augmentation, meditation type, and chills occurrence as factors, with remaining traits and demographics as covariates. All post-hoc comparisons were conducted using Tukey’s Honestly Significant Difference test to control for multiple comparisons. We analyzed the data to isolate the individual contributions of musical augmentation, chills occurrence, and meditation type, examining their distinct and potentially interactive effects.

We additionally computed partial correlations, controlling for demographics and traits, to examine relationships between outcome measures, and a series of mediation and moderation analyses using Jamovi’s *medmod* module (Gallucci, 2020). To test whether the occurrence of chills mediated the relationship between meditation conditions and ST outcomes, we specified a simple mediation model with condition as the predictor variable, chills occurrence as the mediator, and each self-transcendent measure as the outcome variable.

To examine the moderating role of individual trait differences, we conducted several moderation analyses. First, we tested whether *VVIQ* scores moderated the effect of meditation type on connectedness to others by specifying a simple moderation model in *medmod*. We then conducted two separate moderation analyses using *MAIA* scores as the moderator: one examining the relationship between LKM and chills occurrence, and another examining the relationship between chills occurrence and each dependent variable.

For all analyses in *medmod*, we used 5,000 bootstrap samples to estimate indirect effects and generate 95% confidence intervals. Continuous predictor variables were mean-centered prior to analysis. All models included demographic variables and other trait measures as covariates.

### Transparency and openness paragraph

The full dataset is available for download at the following url: https://osf.io/pn4c8/. Stimuli are publicly available on youtube:

LKM(+): https://www.youtube.com/watch?v=Id-zXX7ac-o
LKM(−): https://www.youtube.com/watch?v=R3i3sUywdTo
MC(+): https://www.youtube.com/watch?v=Nxjx6_zYX-4
MC(−): https://www.youtube.com/watch?v=CN-_zzHpcdM

## Results

### Demographics and main effects summary

Participants in each condition showed comparable demographic characteristics (Table 1), though the LKM(−) group showed significantly more liberal/progressive political orientation than the other groups (*F*(3, 216) = 4.426, *p* = 0.005).

**Table 1:** Demographic characteristics by condition.

Condition	Age, M	Age, SD	Gender (M, F, non-binary)	PO, M	PO, SD	Rel., M	Rel., SD	ME, M	ME, SD
MC (+)	36.3	10.5	49, 39, 2	3.86	1.81	19.2	7.87	3.56	1.69
MC (−)	37.4	12.5	49, 46, 4	3.19	1.68	17.5	6.45	3.10	1.52
LKM (+)	34.1	10.5	44, 50, 4	3.21	1.62	17.5	7.26	3.35	1.70
LKM (−)	33.5	11.6	56, 51, 3	2.97	1.61	17.2	7.05	3.31	1.50

MC = mindfulness control; LKM = loving kindness meditation; (+) = with chills augmentation; (−) = without chills augmentation. Rel. = religiosity; ME = meditation experience; PO = political orientation.

Generally, both chills augmentation and meditation type had positive, frequently overlapping effects on the outcome variables, with a general trend toward better outcomes when both factors were combined (Table 2).

**Table 2:** Means and confidence intervals for outcome measures by condition.

Subscale	MC(−) M [95% CI]	MC(+) M [95% CI]	LKM(−) M [95% CI]	LKM(+) M [95% CI]
Arousal post>pre	−0.926 [−1.192, −0.660]	−0.713 [−1.000, −0.426]	−0.808 [−1.078, −0.537]	−0.921 [−1.171, −0.671]
Valence post>pre	0.508 [0.271, 0.746]	0.574 [0.317, 0.830]	0.594 [0.352, 0.836]	0.702 [0.479, 0.926]
Ego-Dissolution	32.0 [29.5, 34.6]	33.9 [31.1, 36.6]	32.8 [30.2, 35.4]	34.9 [32.4, 37.3]
Connection to self	6.04 [5.70, 6.38]	6.43 [6.06, 6.81]	6.36 [6.01, 6.71]	6.26 [5.94, 6.58]
Connection to others	5.68 [5.42, 5.94]	5.83 [5.55, 6.11]	6.47 [6.21, 6.74]	6.44 [6.19, 6.68]
Connection to world/spirituality	4.87 [4.48, 5.26]	5.41 [4.99, 5.83]	5.31 [4.91, 5.71]	5.54 [5.18, 5.91]
Moral elevation	23.4 [22.0, 24.8]	25.4 [23.9, 26.9]	24.8 [23.5, 26.2]	25.6 [24.3, 26.9]
Psychological insight	26.8 [24.3, 29.2]	28.8 [26.2, 31.5]	27.6 [25.1, 30.1]	27.8 [25.5, 30.1]
Emotional breakthrough	22.4 [20.2, 24.6]	25.3 [23.0, 27.7]	24.0 [21.8, 26.2]	24.0 [21.9, 26.0]

Values represent means with 95% confidence intervals in brackets. MC = mindfulness control; LKM = loving kindness meditation; (+) = with chills augmentation; (−) = without chills augmentation.

This general trend was underlied by several hypothesized effects (presented below), with meditation type specifically driving connectedness to others, while chills augmentation and chills occurrence driving the remaining effects.

### Loving-kindness meditation increases connectedness to others

#### Main effects of meditation type, chills augmentation, and chills occurrence

**Figure 1:**
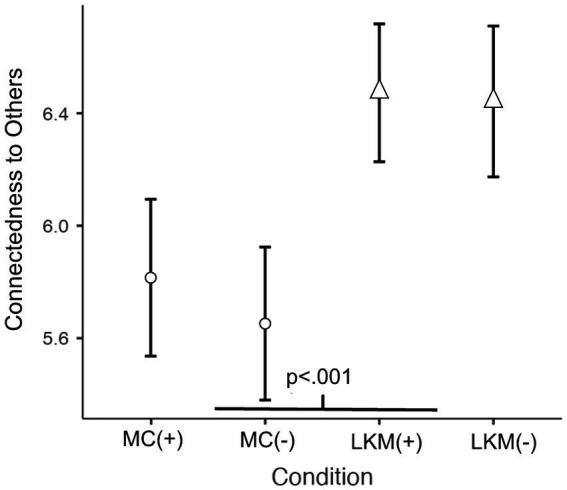
The loving kindness meditation (LKM), regardless of chills augmentation (+/−), caused highly significant increases in connectedness to others (*p* < 0.001) relative to the mindfulness control (MC). Error bars represent 95% confidence intervals.

An ANCOVA revealed Meditation Type as a highly significant predictor of connectedness to others (Figure 1) following intervention (*F*(1, 372) = 28.94, *p* < 0.001). Participants reported significantly higher levels of connectedness to others during LKM compared to the MC condition (Mean difference = −0.721, *SE* = 0.134, *t*(372) = −5.38, *p* < 0.001). Neither the main effects of Chills experience or chills augmentation, nor any of their interactions with Meditation Type were significant (all *p*-values > 0.43).

Trait predictors of connectedness to others included *VVIQ* (*F*(1, 372) = 10.28, *p* = 0.001), *MODTAS* (*F*(1, 372) = 5.19, *p* = 0.023), Agreeableness (*F*(1, 372) = 9.11, *p* = 0.003), and Extraversion (*F*(1, 372) = 4.04, *p* = 0.045).

State predictors of connectedness to others included Pre-Arousal (*F*(1, 372) = 4.96, *p* = 0.027), and Immersion (*F*(1, 372) = 14.99, *p* < 0.001).

### Predictors of chills occurrence

#### Chills augmentation increased self-reported chills

**Figure 2:**
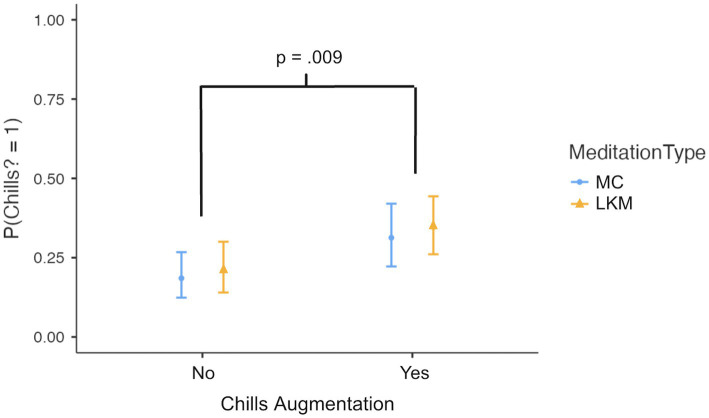
Marginal means for a main-effects-only model. Chills augmentation significantly increased the likelihood of reporting chills across both meditation types (*p* = 0.009), though there was no significant difference between meditation types (*p* = 0.572). Error bars indicate 95% CI.

A logistic regression model (Figure 2) examining main effects predictors of chills occurrence across meditation type and augmentation conditions showed moderate fit (*R*^2^McF = 0.221, AIC = 425). The chills augmentation condition was positively associated with higher likelihood of chills (*b* = 0.695, *Z* = 2.621, *p* = 0.009). *Post hoc* tests found that participants were more likely to report chills during augmented vs. non-augmented meditation (Mean difference = −0.119, *SE* = 0.044, *t*(372) = −2.74, *p* = 0.007, *d* = −0.288). There was no significant difference in chills occurrence between meditation types (Mean difference = −0.027, *SE* = 0.044, *t*(372) = −0.613, *p* = 0.540, *d* = −0.065).

In the MC condition, the probability of experiencing chills increased from 0.185 (95% CI [0.124, 0.267]) without augmentation to 0.313 (95% CI [0.222, 0.420]) with augmentation. Similarly, in the LKM condition, the probability rose from 0.209 (95% CI [0.140, 0.300]) without augmentation to 0.346 (95% CI [0.261, 0.443]) with augmentation.

**Figure 3:**
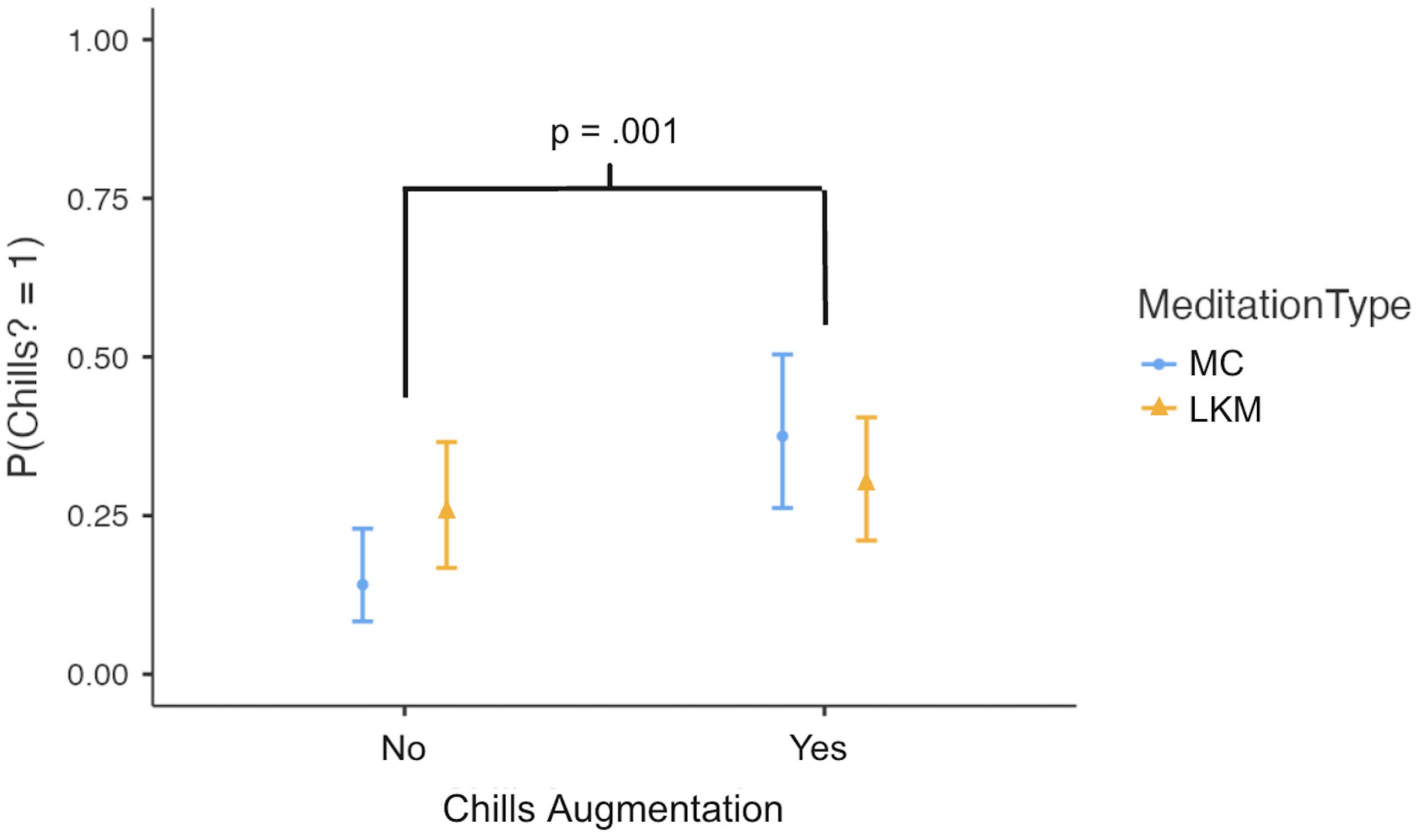
Marginal means after introducing an interaction term. Chills augmentation still significantly increased the likelihood of reporting chills across both meditation types (*p* = 0.001), though the difference between meditation types was somewhat smaller (*p* = 0.064). The meditation type * augmentation interaction was significant (*p* = 0.034), indicating a greater effect of augmentation on the mindfulness control (MC). Error bars indicate 95% CI.

A second more complex model (Figure 3) introducing an interaction term (Meditation Type × Augmentation) found that LKM increased the odds of experiencing chills by 2.07 times compared to MC (*p* = 0.064), while the augmentation condition increased odds by 3.65 times (*p* = 0.001). The significant interaction (OR = 0.34, *p* = 0.042) indicates that these effects were not simply additive. Augmentation was substantially more effective with MC (increasing odds by 3.65 times) compared to LKM (increasing odds by only 1.25 times).

The comparison between models revealed that while both models were significant (*p* < 0.001), Model 2 (with interaction) showed marginally better fit indices overall, with lower deviance (378 vs. 383), lower AIC (422 vs. 425), and slightly higher *R*^2^McF (0.229 vs. 0.221), though a higher BIC (510 vs. 508). The likelihood ratio test indicated that Model 2 represented a modest but significant improvement over Model 1 (χ^2^(1) = 4.20, *p* = 0.040).

Across both models, chills augmentation robustly increased the likelihood of self-reported chills.

#### Predictor covariates of chills

For the more explanatory Model 2, several traits predicted chills occurrence: Conscientiousness demonstrated a significant positive relationship with chills occurrence (*b* = 0.081, *Z* = 2.302, *p* = 0.021) across groups. The analysis also revealed significant positive associations with Kama Muta disposition (*b* = 0.605, *Z* = 3.823, *p* < 0.001).

For state predictors, pre-meditation valence (*b* = 0.296, *Z* = 2.440, *p* = 0.015), and immersion (*b* = 0.599, *Z* = 4.055, *p* < 0.001) were both predictive of chills.

### Chills are associated with self-transcendence

#### Main effects

Chills Augmentation showed significant effects on ego-dissolution (*F*(1, 368) = 4.00, *p* = 0.04, η^2^ = 0.009) and connectedness to world/spirituality (*F*(1, 368) = 5.03, *p* = 0.025, η^2^ = 0.011).

**Figure 4:**
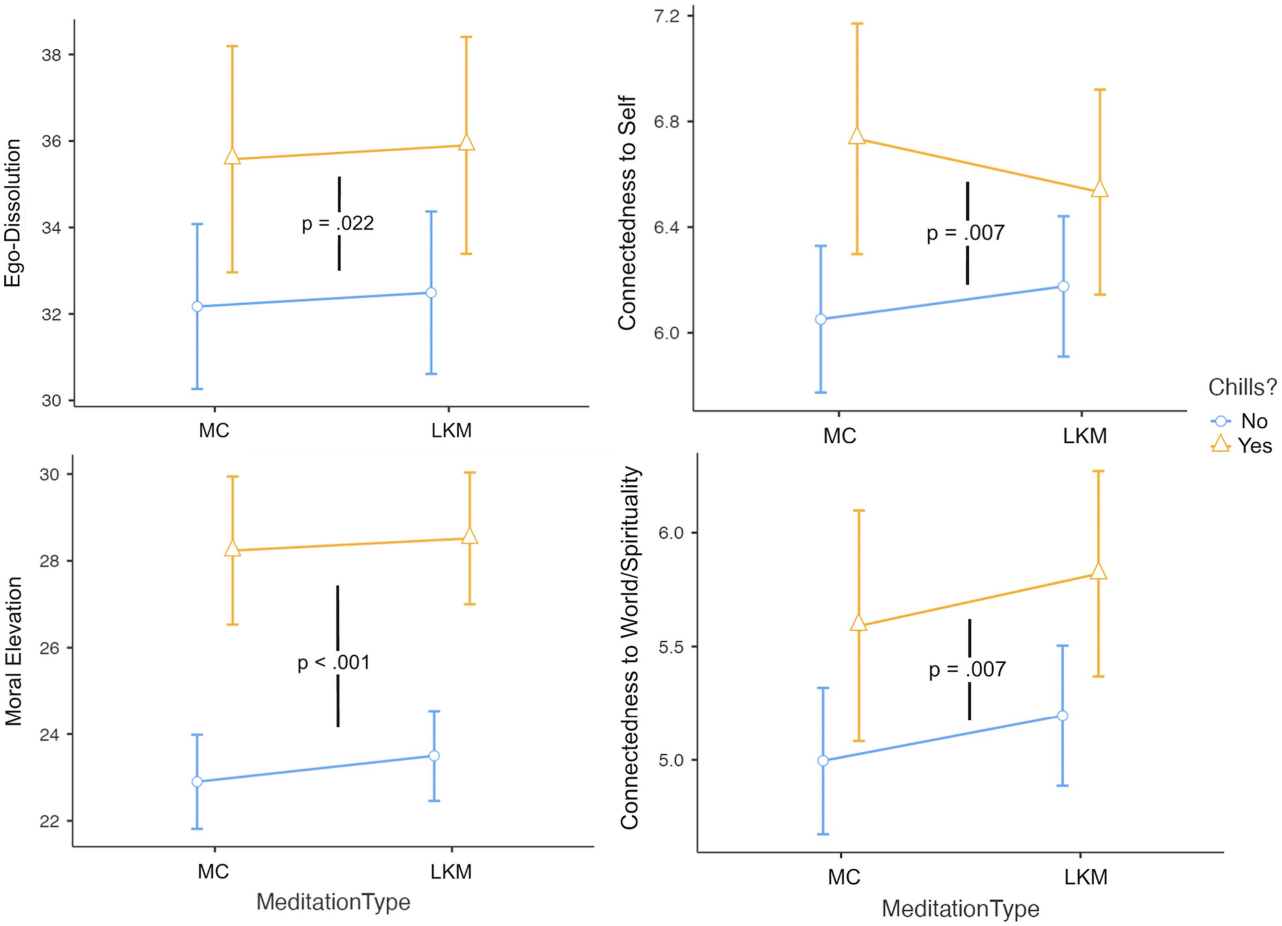
The experience of chills significantly predicted increases in self-transcendence, i.e., ego-dissolution, connectedness to self and world/spirituality, and moral elevation, regardless of meditation type. These were not predicted by meditation type nor its interaction with chills augmentation and chills occurrence. Error bars represent 95% confidence intervals.

The experience of chills emerged as a significant predictor across all measures of self-transcendence: ego-dissolution (*F*(1, 370) = 5.32, *p* = 0.022, η^2^ = 0.011), connectedness to self (*F*(1, 368) = 6.98, *p* = 0.009, η^2^ = 0.015), connectedness to world/spirituality (*F*(1, 368) = 7.54, *p* = 0.006, η^2^ = 0.016), and moral elevation (*F*(1, 368) = 48.52, *p* < 0.001, η^2^ = 0.094). The effect was particularly strong for moral elevation, demonstrating the largest effect size among individual variables (Figure 4).

Notably, neither meditation type nor its interactions with chills augmentation or occurrence were significant predictors for any of these outcome measures (all *p*s > 0.16).

#### Demographic and trait predictors

Trait absorption demonstrated strong effects on multiple outcomes: connectedness to self (*F*(1, 368) = 4.76, *p* = 0.030, η^2^ = 0.010), connectedness to world/spirituality (*F*(1, 368) = 4.00, *p* = 0.046, η^2^ = 0.009), and moral elevation (*F*(1, 368) = 9.61, *p* = 0.002, η^2^ = 0.019). Interoceptive awareness was specifically predictive of connectedness to self (*F*(1, 368) = 6.64, *p* = 0.010, η^2^ = 0.014).

Personality traits showed varying effects. Extraversion predicted connectedness to world/spirituality (*F*(1, 368) = 6.32, *p* = 0.012, η^2^ = 0.014) and moral elevation (*F*(1, 368) = 11.06, *p* < 0.001, η^2^ = 0.022). Conscientiousness predicted connectedness to self (*F*(1, 368) = 5.67, *p* = 0.018, η^2^ = 0.012) and approached significance for connectedness to world/spirituality (*F*(1, 368) = 3.85, *p* = 0.051, η^2^ = 0.008).

Demographic variables showed limited effects, with sex predicting moral elevation (*F*(1, 368) = 7.89, *p* = 0.005, η^2^ = 0.015). Religiosity emerged as a consistent predictor across measures: ego-dissolution (*F*(1, 368) = 5.19, *p* = 0.023, η^2^ = 0.011), connectedness to world/spirituality (*F*(1, 368) = 10.64, *p* = 0.001, η^2^ = 0.023), and moral elevation (*F*(1, 368) = 8.28, *p* = 0.004, η^2^ = 0.016).

#### State predictors

Immersion consistently demonstrated the strongest effects across all measures: ego-dissolution (*F*(1, 368) = 68.61, *p* < 0.001, η^2^ = 0.146), connectedness to self (*F*(1, 368) = 66.50, *p* < 0.001, η^2^ = 0.140), connectedness to world/spirituality (*F*(1, 368) = 48.28, *p* < 0.001, η^2^ = 0.104), moral elevation (*F*(1, 368) = 41.23, *p* < 0.001, η^2^ = 0.080), emotional breakthrough (*F*(1, 368) = 42.90, *p* < 0.001, η^2^ = 0.090), and psychological insight (*F*(1, 368) = 50.37, *p* < 0.001, η^2^ = 0.106).

### Chills are associated with emotional breakthrough and psychological insight

#### Main effects

**Figure 5:**
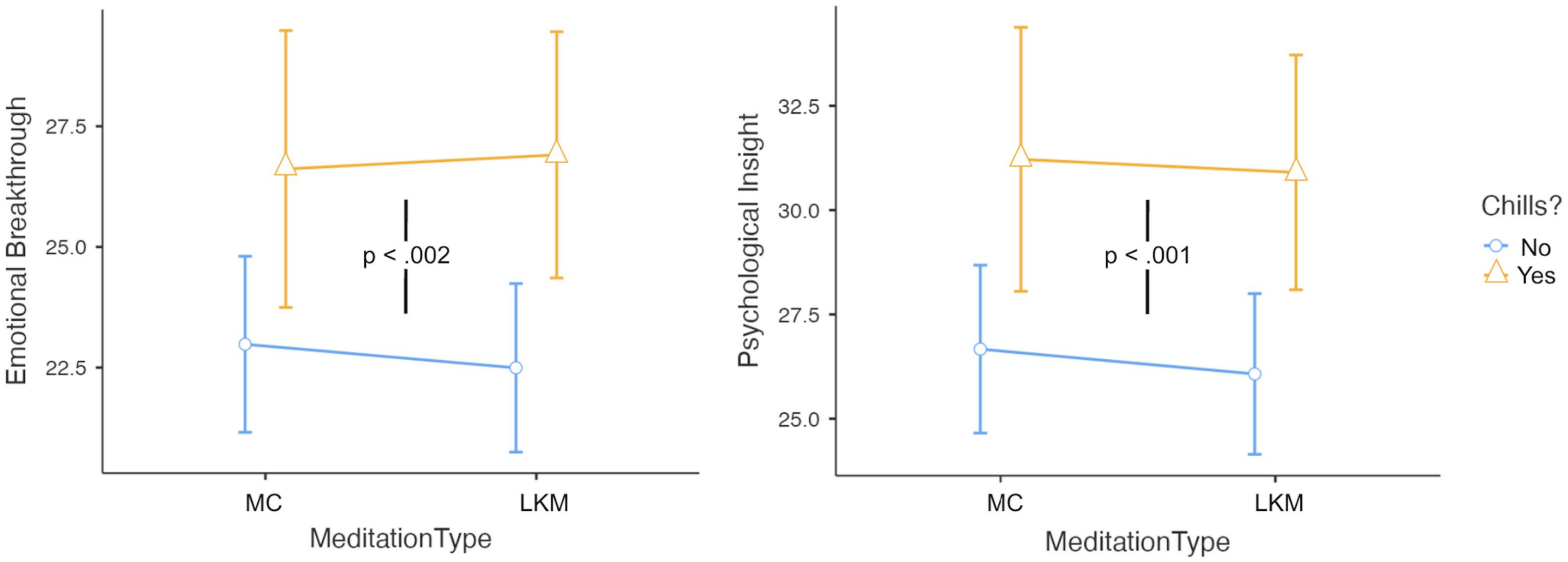
The experience of chills significantly predicted increases in emotional breakthrough and psychological insight, regardless of meditation type. Error bars represent 95% confidence intervals.

The ANCOVAs for Emotional Breakthrough and Psychological Insight (Figure 5) revealed several significant predictors. For both measures, meditation type and its various interactions showed no significant effects (all *p*s > 0.762). The experience of chills, however, was highly predictive of both Emotional Breakthrough (*F*(1, 368) = 11.61, *p* < 0.001, η^2^ = 0.024) and Psychological Insight (*F*(1, 368) = 11.38, *p* < 0.001, η^2^ = 0.024).

#### Trait predictors

Trait absorption emerged as a strong predictor for both measures (Emotional Breakthrough: *F*(1, 368) = 17.73, *p* < 0.001, η^2^ = 0.037; Psychological Insight: *F*(1, 368) = 16.11, *p* < 0.001, η^2^ = 0.034), as did religiosity (Emotional Breakthrough: *F*(1, 368) = 9.28, *p* = 0.002, η^2^ = 0.020; Psychological Insight: *F*(1, 368) = 4.41, *p* = 0.036, η^2^ = 0.009). Additional predictors included Extraversion for Emotional Breakthrough (*F*(1, 368) = 4.08, *p* = 0.044, η^2^ = 0.009) and Age for Psychological Insight (*F*(1, 368) = 4.28, *p* = 0.039, η^2^ = 0.009).

#### State predictors

For both measures, state immersion emerged as the strongest predictor (Emotional Breakthrough: *F*(1, 368) = 42.90, *p* < 0.001, η^2^ = 0.090; Psychological Insight: *F*(1, 368) = 50.37, *p* < 0.001, η^2^ = 0.106).

### Mediation analyses

**Figure 6:**
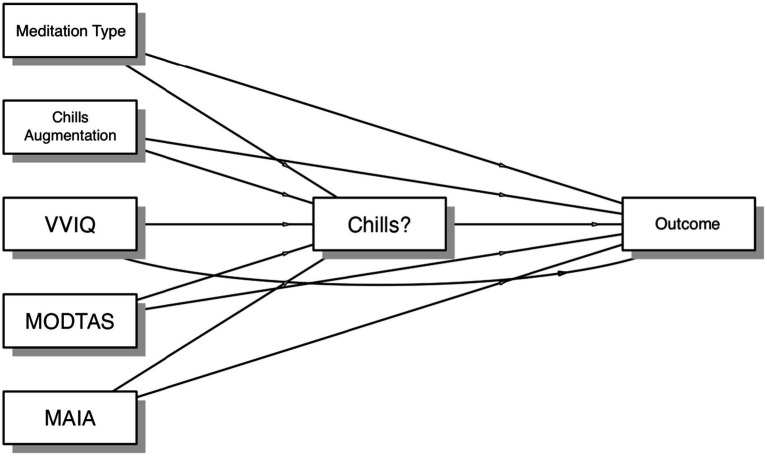
General structure of chills mediation analyses. In each analysis, we examined direct and indirect (chills-mediated) pathways between meditation type, chills augmentation, trait measures of vividness of internal imagery (VVIQ), trait absorption (MODTAS), interoceptive awareness (MAIA), and one of the outcome measures (ego-dissolution, connectedness, moral elevation, emotional breakthrough, psychological insight).

Mediation analyses were conducted to examine the extent to which independent variables and trait differences contributed to outcomes via chills (indirectly) or independently of chills occurrence (directly). Chills occurrence significantly mediated the relationship between both independent variables and traits, and most outcomes except connectedness to others, which among the outcomes was the only one directly influenced by meditation type (see Figure 6).

Meditation type showed direct effects on connectedness to others (*β* = 0.29, *p* < 0.001). Chills augmentation showed indirect effects via chills occurrence for world/spirituality connectedness (β = 0.03, *p* = 0.010; path to chills: β = 0.14, *p* = 0.003), self-connectedness (β = 0.03, *p* = 0.009), moral elevation (β = 0.05, *p* = 0.005), psychological insight (β = 0.03, *p* = 0.010), and emotional breakthrough (β = 0.03, *p* = 0.009).

Chills occurrence directly predicted ego-dissolution (β = 6.77, *p* < 0.001), world/spirituality connectedness (β = 1.08, *p* < 0.001), self-connectedness (β = 0.85, *p* < 0.001), moral elevation (β = 6.38, *p* < 0.001), psychological insight (β = 6.78, *p* < 0.001), and emotional breakthrough (β = 6.38, *p* < 0.001), but not connectedness to others (β = 0.12, *p* = 0.428).

*MODTAS* demonstrated direct effects on ego-dissolution (β = 0.25, *p* < 0.001), world/spirituality connectedness (β = 0.23, *p* < 0.001), self-connectedness (β = 0.25, *p* < 0.001), moral elevation (β = 0.30, *p* < 0.001), psychological insight (β = 0.32, *p* < 0.001), and emotional breakthrough (β = 0.32, *p* < 0.001). Additionally, *MODTAS* showed indirect effects mediated by chills occurrence for ego-dissolution (β = 0.04, *p* = 0.011), world/spirituality connectedness (β = 0.04, *p* = 0.011; path to chills: β = 0.20, *p* = 0.003), self-connectedness (β = 0.05, *p* = 0.002), moral elevation (β = 0.07, *p* = 0.005), psychological insight (β = 0.04, *p* = 0.010), and emotional breakthrough (β = 0.04, *p* = 0.010).

Religiosity showed direct effects on ego-dissolution (β = 0.15, *p* = 0.004), world/spirituality connectedness (β = 0.22, *p* < 0.001), moral elevation (β = 0.19, *p* < 0.001), psychological insight (β = 0.15, *p* = 0.004), and emotional breakthrough (β = 0.21, *p* < 0.001). Its indirect effects through chills occurrence were significant for ego-dissolution (β = 0.03, *p* = 0.044), world/spirituality connectedness (β = 0.03, *p* = 0.043; path to chills: β = 0.13, *p* = 0.026), moral elevation (β = 0.04, *p* = 0.032), psychological insight (β = 0.03, *p* = 0.042), and emotional breakthrough (β = 0.03, *p* = 0.041).

Additional predictors showed direct effects: *VVIQ* on world/spirituality connectedness (β = 0.12, *p* = 0.012; total effect: β = 0.11, *p* = 0.022) and connectedness to others (β = 0.22, *p* < 0.001), and *MAIA* on ego-dissolution (β = 0.10, *p* = 0.045), and self-connectedness (β = 0.22, *p* < 0.001).

Mediation analyses were also conducted on changes to valence and arousal post-intervention, though no significant indirect or direct effects were observed (all *p*s > 0.124).

## Discussion

Our chills augmentation significantly increased the likelihood of experiencing chills and our validated LKM intervention specifically increased feelings of connectedness to others when compared to MC. In a novel finding, chills were found for both meditation types and conditions. Non-augmented mindfulness control and LKM meditations elicited chills in 18.5 and 20.9% of participants, respectively, while chills augmentation increased this likelihood to 31.3 and 34.6%, respectively. Notably, these effects were achieved in a randomly sampled non-expert population with moderate meditation experience on average (on a scale of 1–7, mean = 3.33, SD = 1.61), who were largely able to remain focused throughout the meditation (on a scale of 1–5, mean = 3.51, SD = 1.11). Indeed, meditation experience, while included as a covariate, did not appear to have significant explanatory power with regard to any of the dependent variables (all *p*-values > 0.149), though immersion was strongly implicated in every ANCOVA (all *p*-values < 0.001).

Regardless of condition, the occurrence of chills accompanied increased ST (assayed via ego-dissolution, connectedness to self and the world, and moral elevation), replicating prior findings (Christov-Moore et al., 2024; Schoeller et al., 2023b). The success of the chills augmentation in increasing ST and self-reported emotional breakthrough and psychological insight suggests that chills induced without explicit narrative content (i.e., music chills; cf., Hashim et al., 2023) may be a powerful tool to augment the efficacy and impact of guided narrative-based interventions.

While a significant difference in political orientation was found between the four groups (*F*(3, 216) = 4.426, *p* = 0.005), with the LKM(−) being significantly more liberal, political orientation was included in our analyses to attempt to account for this disparity. We have devoted another recent study to better elucidate the relationship between chills and political orientation, that should lend depth to this observation (Christov-Moore et al., 2025a). Additionally, we noted that the chills augmentation was seemingly more effective for the MC meditation. We speculate that the content of the LKM narrative, which (i) emphasizes a multiscale view of self, (ii) highlights one’s connection to others, and (iii) includes repeated explicit instruction to empathize and convey compassion, may already be inducing a state that the chills-soundtrack can thus only enhance so far (a ceiling effect). This is supported by our past work showing that chills accompany self-transcendence (Christov-Moore et al., 2024).

Independent of the occurrence of chills, individual trait differences selectively predicted variations in effects of the LKM intervention. As hypothesized (hypothesis iv), Vividness of internal imagery (i.e., *VVIQ*) predicted connectedness to others. Additionally, interoceptive awareness (i.e., *MAIA*) and absorption (i.e., *MODTAS*) predicted connectedness to self; absorption and religiosity predicted connectedness to world/spirituality, emotional breakthrough, psychological insight, and moral elevation. *MAIA*, religiosity and absorption predicted ego-dissolution.

The observed relationship between meditation, chills, and enhanced self-transcendence can be understood through the lens of cybernetic control theory (the study of regulation within complex systems), where both meditation and peak experiences enhance psychological flexibility by increasing the degrees of freedom available for information processing and self-regulation (Conant and Ashby, 1970; Safron, 2021). Recent work examining somatic awareness techniques suggests that this flexibility may operate in part through the release of bodily “blockages” that correspond to repressed emotions, with the dissolution of these physical tensions facilitating psychological breakthroughs (Shinozuka et al., 2024). This perspective is supported by neurobiological evidence showing that various nonpharmacological methods of inducing non-ordinary states share common mechanisms with psychedelics (Franco Corso et al., 2023), “encouraging new insights and interpretations” via changes to deep belief structures (Franco Corso et al., 2023). These findings also align with recent theoretical work suggesting that integrated consciousness and self-regulation can be understood as cybernetic control processes operating (and interacting) at different temporal scales (DeYoung and Tiberius, 2023; Goekoop and de Kleijn, 2021).

For example, work on meditation-induced flexibility in self-boundaries (Schweitzer et al., 2024) suggests an optimal balance between sustained practice (cultivating lasting regulatory capacities) and peak experiences (providing moments of enhanced global integration) toward enduring changes in self-processing and social cognition. Here, the synergistic effects of meditation type and chills may reflect an optimal balance of similar complementary mechanisms: meditation types acting on specific long-term control processes, while chills temporarily increase global integration and processing flexibility.

Our findings also complement emerging research on what Yaden et al. (2017) term the “annihilational” and “relational” components of self-transcendent experiences, where reduced self-salience is accompanied by increased feelings of connection to others and one’s environment. Our results suggest that aesthetic chills may operate through similar mechanisms, temporarily dissolving rigid self-boundaries while enhancing feelings of connectedness and prosocial orientation. This aligns with recent work by Schweitzer and colleagues demonstrating that meditation-induced alterations in self-boundaries can enhance prosocial capacities through reduced self-reification, reduced social threat perception, and increased self-other connection. Self-boundary flexibility specifically impacts recognition of negative emotions in others suggesting a shift away from threat-based processing, with our observed effects of chills inducing significant emotional breakthrough and insight (Schweitzer et al., 2024). Such felt sense of boundlessness has also been associated with positive outcomes from psychedelic experience (Goodwin et al., 2025).

Self-transcendent experiences likely exist along a continuum of intensity, ranging from relatively common experiences (e.g., aesthetic chills) to intense, mystical experiences (Schoeller, 2015; Yaden et al., 2017). Our findings that short-lived chills can enhance ST suggest that even transient alterations in self-boundaries can have significant psychological and social effects. ST facilitated by chills augmentation may aid in the translation of other-oriented imagery (engendered by LKM) into prosocial behavior.

While we did not collect neurophysiological data for this study, prior neuroscience research may lend multiple insights into these findings. Chills engage a network of limbic (amygdala and nucleus accumbens) and frontal regions (orbitofrontal cortex and ventromedial prefrontal cortex). Individual differences in chill susceptibility correlate with structural connectivity patterns between auditory association areas, anterior insula, and medial prefrontal cortex (Sachs et al., 2016; Schoeller et al., 2024b). Lesions affecting left insula structural connectivity modulate bodily responses during aesthetic chills while preserving cognitive processing aspects (Witt et al., 2023). The fact that chills lie at the intersection of embodied autonomic processes, affect, and cognition, may explain why chills seem to both mark and potentiate states where the self-boundary (typically tied to embodied signalling) is modulated, and why their intensity and frequency mediates downstream effects on deep beliefs and hedonic patterns (Christov-Moore et al., 2024; Jain et al., 2025; Kähönen, 2023).

Physical manipulation of somatic markers through wearable prostheses enhancing cold sensation can amplify both pleasure and downstream effects of aesthetic chills, substantiating their embodied nature and manifestation within interoceptive/affective/cognitive feedback loops (Haar et al., 2020; Ishikawa et al., 2023). The additional emotional salience of chills-inducing music may further potentiate the relationship of meditative immersion to downstream effects. Chills augmentation thus likely impacts multiple nodes and functional relationships underpinning meditation, its adoption, and its downstream effects.

Recent work from our group has further highlighted the role of interoceptive awareness in chills-occurrence and intensity (Christov-Moore et al., 2025a). The effects of vividness of internal imagery and interoceptive awareness suggests that investigating how meditation and peak experiences interact with individual differences in information processing and self-regulatory capacities could help optimize interventions for different populations (Atasoy et al., 2019; Hoel, 2017). The ubiquitous roles of trait absorption and state immersion in downstream effects is intuitive but also supports the importance of tools to specifically increase immersiveness for intervention efficacy, particularly in novices, and the need to take trait absorption into account when personalizing contemplative interventions and quantifying their effects. These trait and state-related findings usefully suggest multiple avenues and foci for efficacy-boosting personalization of nonpharmacological prosocial interventions (e.g., Schoeller et al., 2024b).

### Limitations

These results and their interpretation are affected by the limitations of survey data: low experimenter ability to control set and setting, deploy implicit, objective measures, and implement more naturalistic measures of prosociality, as well as conduct follow-up studies to assay integration and duration of effect. This is of particular concern in verifying the occurrence of chills directly, as via camera-based assessments of piloerection. However, our group has completed multiple on-site studies of chills (e.g., Christov-Moore et al., 2025b). We make the full current dataset, protocol, and stimuli freely available in the hopes of aiding other groups in replicating and/or extending these studies. One final limitation is that this study focuses on chills occurrence and augmentation with respect to only one of myriad spiritual practices. We are beginning to address this specificity by conducting a study on augmenting prayer practice by pairing it with chills evoking stimuli (Walder et al., 2025).

### Constraints on generality

These results may or may not be specific to characteristics of the participants. The sample is diverse and representative as regards the part of the world being sampled, but does not have equal representation of every subgroup within the sample, nor can it make overly strong claims regarding populations elsewhere in the country, in the world, or outside of WEIRD populations. The experimental context, while not strictly controlled by us as it is a survey study, exists within the same geographical context and is subject to similar constraints and assumptions. In terms of procedures, we are constrained by the explicit or latent idiosyncrasies of the English language, and the conceptual framing used in the briefing.

Regarding stimuli, here we encounter a bit of closed loop: the majority of the stimuli have been sourced by scraping social media (YouTube) for videos that large numbers of users have rated as chills-inducing. Hence, the relationships and effects observed may, and likely do, reflect a matching between the largely WEIRD populations predominating on platforms like YouTube, and the stimuli those populations denote as chills-inducing. While this speaks to the need for matching between subjective aesthetic stimuli and the populations for whom these aesthetics are familiar or compelling, it does not preclude that a similarly matched (using a platform dominated by East Asia, for example) set of stimuli would not elicit the same relationship between chills and characteristics of self-transcendence. Our group is currently running a preregistered study using a subset of the stimuli used here, within a similarly diverse cohort in central Texas. Future studies should examine whether these relationships and effects hold in a progressively wider set of contexts, in other languages, and with a neutral or even misleading briefing. Indeed, the procedure outlined here is in sufficient detail to permit this replication and extension and we hope it will be carried out by other groups, in addition to our future efforts.

## Conclusion

We report the first observation of meditation-induced chills and their strong relation to self-boundary modulation. This further deepens our understanding of both somatic processes in belief-change, and the role of self-transcendent processes in meditative practice and insight. We confirm prior findings showing that loving kindness meditation can reliably enhance prosociality, and that chills mediate many of these effects. Beyond correlational findings, however, musical chills augmentation appears to be able to enhance the self-transcendent and emotionally impactful effects of guided contemplative interventions, without disrupting the effect of the narrative content. Chills-augmented intervention’s effects on impact, insight, and moral elevation aspects demonstrate a likely increase in the likelihood of integration, duration and other-oriented impact of the intervention, noteworthy for the field of empathogenic technodelics.

Future studies should (i) specifically study and target factors affecting immersion, given its importance for outcomes, (ii) further test the association observed between chills as ST by examining horror-related and other negatively-valenced chills, (iii) employ more ecologically valid prosociality measures such as charitable donations, the dictator game and moral dilemmas, (iv) incorporate neurophysiological markers of chills, (v) examine chills augmentation for use in clinical practice with patients experiencing a high degree of rigidity or difficulty when doing mindfulness/LKM practice, in order to increase its benefits, and (vi) see if chills augmentation can enhance specific outcomes of other forms of guided narrative interventions, contemplative and otherwise (e.g., CBT, hypnotherapy).

## Method note

Prolific’s sampling methodology specifically faces several key limitations: the platform experiences rapid-responder bias due to its convenience sampling approach. Though mechanisms exist to distribute study places more evenly among active participants, responses typically come from users who are online when studies launch. The timing of study launches can significantly influence the participant demographic composition. The participant pool exhibits WEIRD bias (Western, Educated, Industrialized, Rich, and Democratic), with data from summer 2018 showing overrepresentation of women, younger individuals, and those with higher education levels. While international in scope, the pool remains predominantly concentrated in the UK and US. Selection bias manifests through the platform’s marketplace structure, where participants can choose studies based on topic interest, reward levels, and time commitments. This self-selection process may result in systematic differences between study participants and the wider population. Finally, the platform faces challenges with reward-motivated participants engaging in satisficing behavior—providing rushed or random responses to maximize earnings per hour. While Prolific implements various screening measures, some of these participants may still enter studies, potentially affecting data quality if not properly identified and removed.

## Data Availability

The datasets presented in this study can be found in online repositories. The names of the repository/repositories and accession number(s) can be found below: the full dataset is available for download at the following url: https://osf.io/pn4c8/. Stimuli are publicly available on youtube: LKM(+): https://www.youtube.com/watch?v=Id-zXX7ac-o&t=961s, LKM(−): https://www.youtube.com/watch?v=R3i3sUywdTo&t=991s, MC(+): https://youtu.be/Nxjx6_zYX-4?si=SdzU1gQAvp41NhVY, and MC(−): https://www.youtube.com/watch?v=CN-_zzHpcdM&t=48s.
